# Documented Caregiver Report of Wheezing at Home is Associated With Decreased Length of Pediatric Asthma Hospitalizations

**DOI:** 10.1177/00099228261461420

**Published:** 2026-06-30

**Authors:** Martha “Grace” James, Katelin Samaniego, Raymond Parlar-Chun

**Affiliations:** 1 McGovern Medical School, Houston, TX, USA; 2 Department of Pediatrics, Children’s Memorial Herman, Houston, TX, USA; 3 Department of Pediatrics, UT Southwestern, Dallas, TX, USA; 4 Children’s Health of Dallas, TX, USA; 5 Department of Pediatrics, McGovern Medical School, Houston, TX, USA; 6 Department of Pediatrics, McGovern Medical School, Children’s Memorial Herman, Houston, TX, USA; 7 Department of Pediatrics, Baylor College of Medicine, Houston, TX, USA; 8 Texas Children’s Hospital, Houston, USA

**Keywords:** asthma, hospitalization, wheezing, pediatric intensive care units, length of stay, caregivers, parents, electronic health records

## Abstract

Recognition of wheezing by parents of children with asthma is often difficult, potentially delaying care and worsening exacerbations. We evaluated whether caregiver report of wheezing at presentation was associated with hospital outcomes. Using chart review of 732 pediatric asthma admissions over 3 years, symptom and demographic data were extracted from the electronic health record, and regression models examined length of stay (LOS) and pediatric intensive care unit (PICU) admission. Wheezing was reported in 45% of cases and was linked to a 10% shorter LOS (IRR 0.9, 95% CI 0.82-0.99, *P* = .04). Commonly reported symptoms included shortness of breath (89%), cough (79%), and congestion (47%). Longer LOS was associated with older age (IRR 1.04, 95% CI 1.03-1.06, *P* < .001) and female sex (IRR 1.14, 95% CI 1.03-1.26, *P* = .01). No variables predicted PICU admission. These findings suggest under-recognition of wheezing by caregivers may contribute to longer hospitalizations and greater exacerbation severity.

## Introduction

Pediatric asthma remains a significant health burden in the United States, affecting approximately 4.5 million children. In 2020, over one-third of children with asthma required emergency department care, while 3.6% necessitated inpatient management, accounting for more than 27 000 hospitalizations.^1,2^ The economic impact is substantial, with direct costs exceeding $278 million annually^3^ in addition to significant indirect costs in lost school and workdays. Continued work to decrease both the frequency and severity of asthma exacerbations is needed.

Early recognition of asthma symptoms by primary caregivers, most often parents, is essential, as timely intervention can prevent progression to severe disease. While signs such as cough and increased work of breathing are easily recognizable, wheezing is harder to identify. Previous studies have shown that parents are poor at labeling their child’s respiratory sounds as wheezing.^4^ They also have inconsistent definitions and understanding of wheezing, and prior work has shown that fewer than 25% of parents will self-report wheezing as a symptom of asthma.^4-6^ It is unknown if primary caregivers’ ability to correctly recognize wheezing is associated with better outcomes for their children during an acute attack.

This study examines whether caregiver reports of wheezing as documented in the history of present illness (HPI) are associated with hospitalization outcomes in children with asthma exacerbations. We hypothesize that documented wheezing as a proxy for correct or early identification of an asthma exacerbation is associated with decreased severity of exacerbations, leading to decreased length of hospitalization (LOS) and decreased risk for pediatric intensive care unit (PICU) admissions.

## Methods

### Population

This was a single-site retrospective cohort study of pediatric patients admitted to an urban tertiary hospital with asthma exacerbation over a 3-year period. Asthma exacerbations were screened using International Classification of Diseases (ICD) discharge codes (493.x) and then confirmed by chart review by the principal investigator. Patients less than 4 years old were excluded to homogenize the population from competing diagnoses such as bronchiolitis, wheezing-associated respiratory illness, or reactive airway disease. Adult patients (18 and older) were not included in this patient population. Patients with home mechanical ventilation and the use of tracheostomy were also excluded.

### Data From the Electronic Medical Record

The initial intake history and physical (H&P) for each hospitalization was pulled from the electronic health record (EHR), and the HPI section was analyzed. All reported symptoms from the HPI were recorded and verified by 2 members of the study team. Similar symptoms were grouped by clinical relevance and total counts were tabulated. The words “wheeze” or “wheezing” had to be directly documented in the HPI to count wheezing as a reported symptom.

In addition to description of symptoms, other variables collected for each admission were age, sex, prior history of asthma, duration of first symptoms in days before hospitalization, and the use of albuterol before medical care. Prior history of asthma was indicated as positive if asthma history was documented in either the history of present illness or past medical history of the H&P. Duration of first symptoms was calculated based on the onset of first reported symptom from the HPI. This was dichotomized to less than 24 hours and greater than 24 hours to investigate associations of acute onset of symptoms. Use of albuterol was defined as positive for any reported albuterol use before arrival at the emergency department.

### Outcomes and Analysis

We used LOS and PICU admissions as measures of asthma severity outcomes. Length of stay (LOS) was defined in hours as the duration of time from arrival to a hospital bed to discharge from the hospital. Given the nonnormal distribution, this was treated as a count and modeled using a negative binomial regression model. Pediatric intensive care unit admission was defined as a binary outcome and analyzed using a logistic regression model. Both models were adjusted for clinically relevant variables.

Descriptive statistics were used to characterize our population and reported as mean (SD), median (interquartile range [IQR]), or count (%) as appropriate. *P* values of less than .05 were considered significant. Analysis was performed using Stata v.15. Local institutional review board (IRB) approval with our institution was obtained with a waiver of consent.

## Results

Overall, 732 patients met criteria to be included in our cohort. The average age was approximately 8 years with 63% males and 37% females. A total of 605 (83%) were hospitalized with a known history of asthma, and 509 (70%) had used albuterol before presenting to the emergency department. 371 (51%) had onset of reported symptoms less than 24 hours before presentation to the emergency department. When described by reported wheezing status, we find that the baseline characteristics of the 2 groups to be similar (Table 1).

**Table 1.** Characteristics of Cohort.

	Wheezing reported n = 333 (45%)	Wheezing not reported n = 399 (55%)
Age (years)	7.9 (3.3)	7.8 (3.3)
Sex		
Male	202 (61%)	257 (64%)
Female	131 (39%)	142 (36%)
Albuterol use before the emergency room		
Yes	235 (71%)	274 (69%)
No	98 (29%)	125 (31%)
Known history of asthma		
Yes	276 (83%)	329 (82%)
No	57 (17%)	70 (18%)
Duration of symptoms before admission		
Less than 24 hours	170 (51%)	191 (48%)
More than 24 hours	163 (49%)	208 (52%)
Length of hospitalization hours (SD)	50 (44)	54 (37)
PICU admission	33 (10%)	53 (13%)

The most reported symptom was clinical signs of respiratory distress with 653 (89%), reported as shortness of breath or increased work of breathing. Another commonly reported symptom was cough with 580 (79%). Less than half of caregivers reported wheezing 333 (45%). Table 2 describes the top 10 reported symptoms.

**Table 2.** Most Commonly Reported Symptoms.

1	Shortness of Breath/Work of Breathing	653 (89%)
2	Cough	580 (79%)
3	Rhinorrhea/Congestion	342 (47%)
4	Wheezing	333 (45%)
5	Fever	168 (23%)
6	Vomiting	151 (21%)
7	Chest pain/tightness	111 (15%)
8	Decreased Activity or Intake	93 (13%)
9	Sore Throat	47 (6%)
10	Abdominal Pain	36 (5%)

We find a protective association with caregiver reporting of wheeze, with patients spending 10% less time in the hospital (IRR 0.9, 95% CI 0.82-0.99, *P* = .04). Other significant associations with LOS were age, with each additional year of life increasing LOS by 4% (IRR 1.04, 95% CI 1.03-1.06, *P* < .001) as well as females having 14% increased LOS compared with males (IRR 1.14, 95% CI 1.03-1.26, *P* = .01). No significant associations with LOS with albuterol use before presentation to the ER, prior history of asthma, or onset of symptoms within one day of presentation (Table 3).

**Table 3.** Association of Reported Wheezing and Length of Hospitalization.

	IRR	95% CI	*P* value
Caregiver report of wheeze	0.9	0.82-0.99	.04
Age (years)	1.04	1.03-1.06	<.001
Female	1.14	1.03-1.26	.01
Albuterol use before ER	1.04	0.93-1.17	.46
Known history of asthma	0.9	0.78-1.03	.13
Duration of Symptoms < 24 hours	1.05	0.95-1.16	.35

Although the point estimate suggests a decrease in the odds of PICU admission for patients whose caregivers reported wheezing, this association was not statistically significant. None of the other variables were associated with PICU admission (Table 4).

**Table 4.** Association of Reported Wheezing and PICU Admission.

	OR	95% CI	*P* value
Caregiver Report of Wheeze	0.71	0.45-1.27	.15
Age (years)	1.05	0.98-1.12	.14
Female	1.23	0.78-1.96	.38
Albuterol Use Prior to ER	0.76	0.46-1.27	.29
Known History of Asthma	1.38	0.69-2.8	.36
Duration of Symptoms <24 hours	0.91	0.58-1.45	.7

## Discussion

In our cohort of children admitted for acute asthma exacerbations, we find that patients with caregiver-reported wheezing had an associated 10% decrease in LOS compared with those whose caregivers did not report wheezing. Furthermore, wheezing was reported in only 45% of intake histories. There was no statistically significant association in PICU admissions among patients with caregiver-reported wheezing.

Our results support the hypothesis that primary caregiver recognition of wheezing leads to less severe asthma exacerbations, secondary to earlier and more accurate recognition of symptoms. If true, educational interventions in the clinics or hospitals that teach primary caregivers how to recognize wheezing may help decrease health care use. Prior studies have shown that short education sessions demonstrating auscultation techniques can improve recognition of obvious wheezes, although subtle wheezes may still be missed.^7^ Digital technologies, such as mobile apps or wheeze detection devices, may also help caregivers recognize wheezing. While these devices have shown promise, further studies are needed to confirm their efficacy.^8^ When creating educational content for caregivers, it is important to objectively define wheezing, as distinguishing it from other respiratory sounds can be a difficult task even for trained medical providers.^6^

Although wheezing is a universal asthma symptom, it was reported in less than half of the intake histories. This contrasts with the proportion of caregivers that reported cough (79%) and shortness of breath or increased work of breathing (89%). This highlights the large disparity in the recognition and reporting of wheezing, which may lead to missed opportunities for timely intervention. Our results are similar to previous studies. In a cross-sectional study involving 200 parents of children with and without asthma, only 38% were able to identify wheezing from video clips of respiratory sounds.^4^ In another study, less than 25% of parents reported wheezing as a sign of acute asthma.^6^ Clearly, more work is needed to educate families on recognizing wheezing.

It is also important to recognize the dynamic nature of wheezing during asthma exacerbations. Children with severe obstruction may initially lack wheezing, a phenomenon known as “silent chest”, which can later progress to audible wheezing as airflow improves with or without treatment.^9^ In other cases, wheezing may resolve quickly with bronchodilators. These fluctuations in wheezing make it difficult to interpret caregiver symptom reports taken at a single time point and may partially explain the low rates of wheezing documentation in our study.

Despite the association with decreased length of stay, we did not observe a statistically significant reduction in PICU admissions. The point estimate of a 30% protective effect is promising, and we hypothesize that the lack of statistical significance is likely due to the low event rate of ICU admissions and the lack of power in our study to capture this. Further studies are necessary to observe the relationship between caregiver recognition of wheezing and the need for intensive care.

Our results should be interpreted in the context of various limitations. First, this was a single-center study at an urban tertiary care hospital, which limits generalizability in other settings. We recognize that length of hospitalization, decision to hospitalize, and decision to admit to the PICU are institution-dependent. In addition, caregiver-related factors may impact the accuracy of reported wheezing. We did not collect data on the caregiver such as age, relationship to child, hearing ability, extent of caregiving responsibilities (eg, caring for multiple children), occupation, all of which could affect their ability to detect symptoms. It is also likely that some caregivers recognized wheezing before presentation; however, they failed to mention when being asked about the child’s symptoms.

Next, our observation of reported wheezing documentation is likely an underestimation of the true rate of caregiver recognition of wheezing. Given the retrospective nature of our study, we were unable to control for the skill level of physicians performing the original history or use a standardized questionnaire to ensure that all providers were asking about wheezing. Physicians may not have directly prompted this symptom, or the documentation itself may not have been accurately transcribed. Nevertheless, the large difference in documentation of 2 universal symptoms of asthma exacerbations, shortness of breath (89%) and wheezing (45%), indicates to us that there is indeed a true under-recognition of this symptom.

Lastly, while our study controlled for some important clinical factors associated with hospital length of stay, we did not control for various factors previously identified in the literature. This includes factors such as but not limited to children with complex chronic conditions, weekend and seasonal admissions, co-morbid psychiatric conditions, and patient socioeconomic status.^10-13^ We were also unable to objectively measure severity of illness on presentation, as our hospital does not use a standardized or validated asthma severity scoring system. Established tools, such as the Pediatric Assessment Triangle (PAT) and pulmonary score (PS), incorporate vital signs on presentation and are used to predict the need for hospitalization in pediatric asthma.^14^ Higher scores on the PAT and PS have been associated with longer stays in the emergency department. Without these measurements at presentation, our study needed to use the length of hospitalization and the need for ICU admission as proxies for severity. However, we recognize these outcomes may be influenced by institutional practices and may not consistently reflect true illness severity, given that both groups had similar lengths of stay. Further analysis that stratifies patients based on the severity of their clinical symptoms at presentation is warranted.

## Conclusion

Caregiver-reported wheezing is associated with shorter hospitalization in children with asthma exacerbations. Educational interventions aimed at improving caregiver recognition of wheezing may facilitate earlier intervention, reduce hospital use, and improve clinical outcomes. Further research should explore specific caregiver education strategies and their impact on wheezing recognition.

## Author Contributions

Dr. Parlar-Chun conceptualized the study, collected data, analyzed data, contributed to manuscript writing, and revised and approved the final manuscript.

Dr. James collected data, led manuscript writing, and approved the final manuscript.

Dr. Samaniego collected data, revised and approved the final manuscript.

## Data Availability

To protect the privacy of research participants, data are not publicly available.
